# Baduanjin Exercise for Stroke Rehabilitation: A Systematic Review with Meta-Analysis of Randomized Controlled Trials

**DOI:** 10.3390/ijerph15040600

**Published:** 2018-03-27

**Authors:** Liye Zou, Chaoyi Wang, Xiaoan Chen, Huiru Wang

**Affiliations:** 1 Department of Sports Science and Physical Education, The Chinese University of Hong Kong, Hong Kong, China; 2 College of Physical Education, Jilin University, Changchun 130012, China; chaoyiw@gmail.com; 3 College of Sports Science, Jishou University, Jishou 416000, China; Shenxiaoan@jsu.edu.cn; 4 Department of Physical Education, Shanghai Jiaotong University, Shanghai 200240, China

**Keywords:** Baduanjin, mind-body exercise, stroke, rehabilitation, balance function

## Abstract

*Objective*: The purpose of this review was to objectively evaluate the effects of Baduanjin exercise on rehabilitative outcomes in stroke patients. *Methods*: Both Chinese and English electronic databases were searched for potentially relevant trials. Two review authors independently screened eligible trials against the inclusion criteria, extracted data, and assessed the methodological quality by using the revised PEDro scale. Meta-analysis was only performed for balance function. *Results*: In total, there were eight randomized controlled trials selected in this systematic review. The aggregated result of four trials has shown a significant benefit in favor of Baduanjin on balance function (Hedges’ g = 2.39, 95% CI 2.14 to 2.65, *p* < 0.001, *I*^2^ = 61.54). Additionally, Baduanjin exercise effectively improved sensorimotor function of lower extremities and ability of daily activities as well as reduced depressive level, leading to improved quality of life. *Conclusion*: Baduanjin exercise as an adjunctive and safe method may be conducive to help stroke patients achieve the best possible short-term outcome and should be integrated with mainstream rehabilitation programs. More rigorous randomized controlled trials with long-term intervention periods among a large sample size of stroke patients are needed to draw a firm conclusion regarding the rehabilitative effects for this population.

## 1. Introduction

Stroke (also called cerebrovascular disease) is a devastating medical condition in which cerebral vascular dysfunction suddenly occurred, leading to cell deaths [1]. This neurological disorder is caused by cerebral blood flow and cerebral vascular occlusion (ischemic stroke) or cerebrovascular rupture (hemorrhagic stroke) [2]. Sensorimotor impairments (e.g., losses of body sensation and proprioception, and altered patterns of coordination and balance) are commonly reported among survivors of stroke, which is directly associated with inability to initiate voluntary movement or hemiplegia [3]. In addition, depressive symptom is a common and serious complication among post-stroke survivors; roughly 30% of post-stroke survivors develop depression, which may be attributed to feeling hopeless about their future lives [4]. Such stroke-induced symptoms have greatly affected activities of daily living and reduced quality of life [5,6].

Globally, 15 million individuals suffer from a stroke each year, with 5.5 million deaths and 5 million with a loss of their capabilities for independent living [7]. An epidemiologic study published in 2007 reported that the number of stroke cases rapidly increased in China and had reached more than 7 million; roughly 70–80% of individuals survive who lost functional abilities and require treatment to improve the stroke-related symptoms [8]. In the United States, more than 700,000 individuals suffer a stroke annually and roughly two-thirds of these post-stroke survivors require rehabilitation [9]. Available evidence indicates that conventional rehabilitation techniques (cycle-ergometer, rising from a chair) and device-assisted therapy (virtual reality) are effective for post-stroke survivors [10,11]. However, it is noted that these rehabilitation methods are time-consuming and costly, which may preclude post-stroke survivors with low social-economic status [12]. In addition, these rehabilitations have simply emphasized physical training for improving sensorimotor function; the mental illness (depression, anxiety, and mood disturbance) in post-stroke survivors could not be alleviated. Therefore, searching for more cost-effective and readily accessible approaches is critically demanded for improving both physical and psychological symptoms among post-stroke survivors.

Baduanjin, as one of the traditional Chinese health-promoting exercises (e.g., Tai Chi, Baduanjin, Wuqinxi), was originally created to help soldiers recover from bodily injuries in ancient China and prepare for the next battle [13,14,15]. Baduanjin contains eight simple postures and movements, which makes it readily accessible to a variety of people [16]. Additionally, when compared to other conventional physical exercises, Baduanjin training does not simply improve musculoskeletal and neuromuscular function [17], but its unique features (mind-body relaxation, mental focus, and breathing control) may also make post-stroke survivors feel more pleasant and enhance their adherence to Baduanjin exercise for psychological well-being [18]. Baduanjin has become more popular since the in 2002 Chinese Health-Qigong Association was established in 2002 to introduce this mindful exercise to people around the world, which has naturally attracted attention from the research community [19]. Although the number of trials were conducted to investigate the effects of Baduanjin exercise on rehabilitative outcomes in post-stroke survivors [20,21,22,23,24,25,26,27], to date no systematic review has been done to synthesize these research findings. Thus, we conducted a systematic review to objectively evaluate the existing literature relating to the rehabilitative effects of Baduanjin for post-stroke survivors. The updated information of this review would allow future researchers and clinicians to design and develop effective mind-body exercise protocols for accelerating the psychosomatic rehabilitation of post-stroke survivors.

## 2. Methods 

### 2.1. Data Sources 

We used the following databases for literature search without restricting date of publication: PubMed, Cochrane Library, WHO International Clinical Trials Registry Platform, Science Direct, Allied and Complementary Medicine Database, China National Knowledge Infrastructure, Wanfang, and Chinese Clinical Trial Registry. To ensure inclusion of all relevant trials, relevant search terms were combined with Boolean conjunction (OR/AND) and used according to three search levels: (i) Baduanjin or Eight Brocade Section; (ii) stroke, cerebrovascular accident, or cerebrovascular disease; (iii) rehabilitation, therapy, balance, functional status, sensorimotor function, or mental disorder. Cross-reference search was also used to manually identify relevant trials. The detailed information of this systematic review and meta-analysis is reported following the Preferred Repointing items for Systematic Reviews and Meta-Analyses (PRISMA) guidelines [28].

### 2.2. Inclusion Criteria and Study Selection

Randomized controlled trials were published in a peer-reviewed English or Chinese journal and investigated the rehabilitative impacts of Baduanjin for stroke survivors. Trials in which there was at least one pairwise comparison between an experimental group that received Baduanjin exercise as the main intervention and a passive (unaltered lifestyle or waitlist) or active control (walking, drug therapy, or balance training) group. Rehabilitative outcomes were considered in this systematic review, including sensorimotor function, balance, abilities of daily living, and psychological parameters (e.g., depression or anxiety), and quality of life. Documents excluded involved observational studies, case studies, case reports, unpublished thesis and dissertation, and conference proceeding.

### 2.3. Methodological Quality Assessment of Eligible Studies

The revised Physiotherapy Evidence Database (PEDro) scale was used to assess the study quality of trials selected [29]. The revised PEDro contains 10 items: random assignment, concealed allocation, similar baseline, blinding of assessors, more than 85% retention, missing data management, between-group comparison, point measure and measure of variability, isolated Baduanjin intervention, and prior sample size calculation. Blinding of participants and instructor(s) were not considered because they are impractical in exercise intervention studies. Each study can obtain a sum score ranging from zero to ten, with higher scores indicating better study quality.

### 2.4. Data Extraction and Analysis 

All identified trials were reviewed by two independent authors (Liye Zou and Chaoyi Wang). The detailed information from eligible trials was extracted, including reference (name of leading author, year of publication, and type of experimental design), location (language), participant characteristics (sample size and dropout rate, mean age/age range of study participants, course of disease, and type of stroke), intervention program (training duration and intensity), outcome measured, study findings, adverse event, and follow-up assessment. Because the heterogeneity of outcome measures in a small number of randomized controlled studies, meta-analysis (Comprehensive Meta-Analysis Version 2.0 software; Biostat, NJ, USA) was only performed on balance function. While the fixed-effect model was used, effect size (Hedge’s g) was used to evaluate the magnitude of the effect of Baduanjin on balance function in stroke patients. The value of Hedges’ g can be interpreted based on the following rule of thumb: small effect = 0.2, medium effect = 0.5, and large effect = 0.8 [30].

## 3. Results

### 3.1. Study Selection

**Figure 1:**
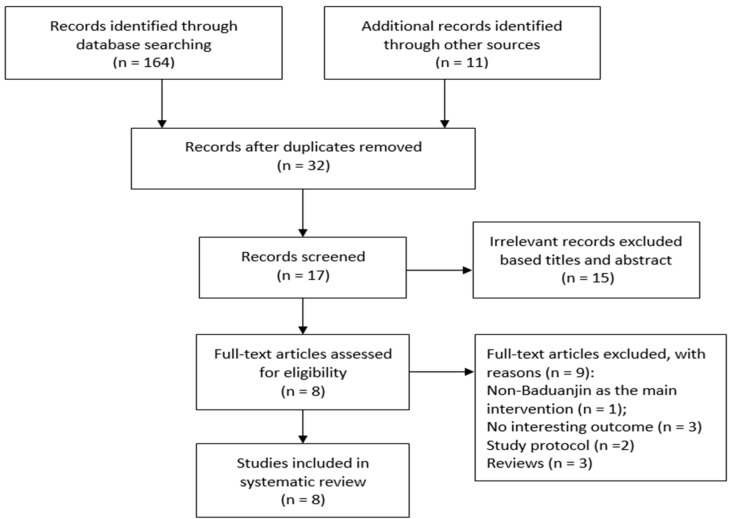
The flow of our literature search and selection process.

Literature search resulted in 175 potentially relevant articles. After removing 143 duplicates, 32 records remained. When reading the titles and abstract of the remaining studies, 15 irrelevant records were identified and removed. This is followed by full-text article evaluation and nine full-text articles were excluded (non-Baduanjin as the main intervention = 1; reviews = 3; No interesting outcome = 3; study protocol = 2). Finally, a total of 8 randomized controlled trials was left in this systematic review (Figure 1).

### 3.2. Study Characteristics

Eight randomized controlled trials were conducted between 2010 and 2017 in China. All trials in which there were 822 older adults with stroke (sample size in individual trials ranging from 60 to 224). Course of disease among these post-stroke survivors was reported ranging from one to six months. In total, there were 269 participants with ischemic stroke and 105 with hemorrhage stroke in five trials (other two trials did not report the type of stroke). Baduanjin intervention program (training duration ranged from 40 days to 12 weeks) varied from 30 to 80 min per day, two to seven times weekly. Group-based Baduanjin training was carried out in all clinical trials by certified instructors. Baduanjin as the main intervention was combined with educational program, general rehabilitation, Bobath technique and drug therapy, or balance training and general rehabilitation. Post-stroke patients in the active control groups had received educational program, general rehabilitation, Bobath technique and drug therapy, or balance training and general rehabilitation (Table 1).

### 3.3. Study Quality 

The methodological quality of all trails selected ranged from five to seven, with a higher score indicating lower risk of bias (Table 2). Points in all trials were deducted due to the lack of concealed allocation and isolated Baduanjin intervention and prior sample size estimation. This is followed by deducted points on blinding of assessors in five trials [20,24,25,26,27] and missing data management in three trials [24,25,26].

### 3.4. Effects of Baduanjin on Stroke-Related Outcomes 

**Figure 2:**
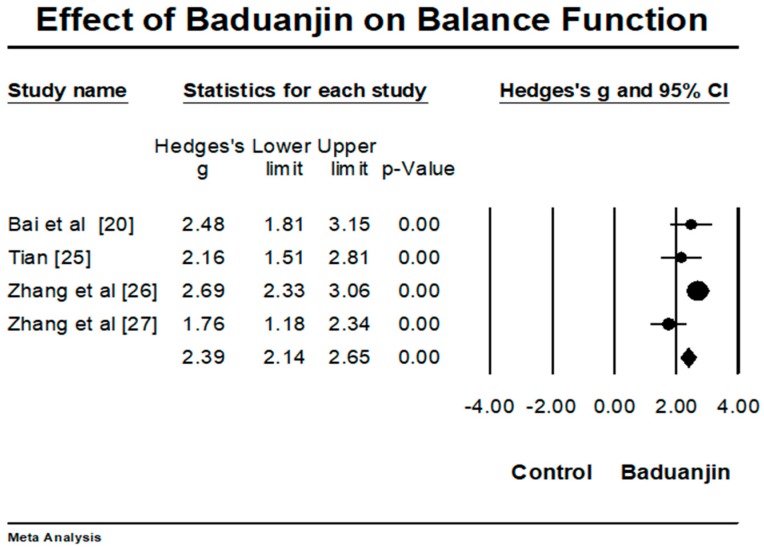
Effect of Baduanjin on balance function.

For meta-analysis, four trials investigated effects of Baduanjin on balance function, as measured by the Berg Balance Scale [20,25,26,27]. A higher positive value indicates better balance function. The aggregated result has shown a significant benefit in favor of Baduanjin on balance function (a large effect size, but moderate heterogeneity: Hedges’ g = 2.39, 95% CI 2.14 to 2.65, *p* < 0.001, *I*^2^ = 61.54) (Figure 2).

Sensorimotor function and neurological deficit: two trials assessed sensorimotor function on lower extremity with the Fugl-Meyer Assessment-Lower Extremity [25,27]. Study findings from the two trials indicated significant improvement on lower extremity-related sensorimotor function. Specifically, Tian et al. [25] considered Baduanjin + general rehabilitation as an experimental group, whereas general rehabilitation alone was thought of as a control group. After a 12-week intervention period, Baduanjin group (12.45 ± 7.24 vs. 23.31 ± 7.63) showed significantly greater improvement on sensorimotor function of lower extremities than the control group (13.37 ± 6.55 vs. 17.13 ± 5.72). Another trial in which researchers compared an experimental group (Baduanjin + balance training + general rehabilitation) with a control group (balance training + general rehabilitation) suggested that Baduanjin (12.3 ± 3.56 vs. 27.18 ± 5.11) also had significantly greater improvement on sensorimotor function of lower extremity than the control group (12.44 ± 3.2 vs. 21.17 ± 4.36) [27]. Additionally, Guo et al. [24] investigated the effects of Baduanjin on neurological function, as measured by National Institute of Health Stroke Scale (a higher score indicating worse symptom), suggesting that Baduanjin group (15.97 ± 2.84 vs. 5.25 ± 2.54) showed significantly greater improvement than the control group (16.17 ± 2.76 vs. 7.66 ± 2.49).

Depression, activities of daily living, and quality of life: two trials assessed quality of life with two different self-reported questionnaires [20,23]. Cai [20] reported that significant improvements on physical, psychological, and environmental domains of World Health Organization Quality of Life were only observed in Baduanjin group (*p* < 0.05) from baseline to Week-12, but not in the control group. Another trial by Chen et al. [23] in which both groups received Baduanjin practice combined with different components (general rehabilitation vs. general rehabilitation + music) suggested that significant improvement on the sum score of quality of life, as measured by the SF-36. As compared to the general rehabilitation group, Baduanjin with music was more beneficial for improving quality of life and eight individual domains (vitality, physical functioning, bodily pain, general health perceptions, physical role function, emotional role functioning, and mental health) [23]. Meanwhile, Chen et al. [23] also assessed depressive symptom (with the Hamilton Depression Scale) among post-stroke survivors, suggesting that Baduanjin effectively reduced depression in both groups, with greater reduction of depression in a combined group (Baduanjin + music + general rehabilitation). The ability of daily activities was assessed using the Barthel Scale in a trial in which an experimental group receiving a combination of Baduanjin and an education program was compared with those people in a control group who received the same educational program alone [22]. Cai et al. [22] reported significant improvement in the Barthel Scale in the Baduanjin group (*p* < 0.001), but not in the control group.

## 4. Discussion

The purpose of this systematic review was to critically evaluate the effects of Baduanjin exercise on rehabilitative outcomes among post-stroke survivors. The study findings from this present review suggest that Baduanjin exercise may be a promising approach for improving functional capabilities of lower extremities (static and dynamic balance and sensorimotor function) and alleviating depressive symptoms and reducing neurological deficit scores. Baduanjin exercise may be also conducive to improving quality of life and the ability of daily activities among survivors of stroke.

Baduanjin routine involves symmetrical postures and coordinated movements, which requires practitioners to sustainably maintain the balance with weight-shifting movement or moving their arms, legs, and torso to change the center of gravity. For example, in performing Movement 5 (“sway the head and shake the tail”), the practitioner needs to squat in a low horse stance while placing the hands on the thighs with the elbows facing out, followed by drawing a circle with his or her upper body (shift his or her weight of the upper body on each side) without losing his or her balance. Thus, it is reasonable that Baduanjin exercise has a significant effect on improving balance function and sensorimotor function of lower extremities among post-stroke survivors. Such these study findings of the present review are supported by previous studies investigating the rehabilitative effects of Baduanjin for functional abilities of lower extremities in vulnerable populations, particularly in patients with Parkinson’s disease who are typically facing balance function impairment [31,32,33]. Baduanjin does not only focus on strengthening physical function, but also has emphasized mental practice to coordinate with breathing technique and musculoskeletal relaxation. Chan et al. (2017) found that mindfulness-based Baduanjin exercise effectively elevated adiponectin level, which potentially contributed to reduced depression in patients with chronic disease [34]. It may be a reason the depressive symptom in post-stroke survivors was alleviated. Since these aforementioned outcomes were improved, the ability of daily activities and quality of life of post-stroke survivors may be improved accordingly.

Despite the promising results were found in the present review, several limitations must be acknowledged. First, all trials selected had a higher risk of bias in this systematic review, which is due to lack of concealed allocation, blinding of assessors, missing data management, and prior power analysis. In particular, one point was deducted in all trials because Baduanjin exercise was combined with other mainstream rehabilitation programs, meaning that it may be difficult to determine whether the positive outcomes were due to Baduanjin alone or synergetic effects. It must be admitted that combined intervention is legitimate because the ultimate goal of Baduanjin exercise as an adjunctive method is to help survivors become as independent as possible and to attain the best possible quality of life. Second, two-thirds of the trials selected involved short duration of intervention without follow-up assessment, so the study findings from the existing literature are limited to the immediate effects of Baduanjin. Third, because of the limited number of studies investigating the effects of Baduanjin on sensorimotor function and neurological function, the ability of daily activities, and quality of life, it remains unclear whether these outcomes were improved by chance. Fourth, all trials were conducted in China and the stroke patients were predominantly Chinese. It is unclear whether the improved balance is generalizable to non-Chinese populations. Fifth, although meta-analysis with RCTs is advantageous, given the fact the study design allows for the determination of the effect of an intervention, it actually decreases the pool of studies from which the authors could gather data; in particular, only 8 articles were included in the current study. For example, other experimental studies (e.g., controlled trials with no randomization and pre-test and post-test studies) may be able to partially explain the relationship between Baduanjin exercise and rehabilitative outcomes.

To better evaluate the effects of Baduanjin exercise on rehabilitative outcomes in patients with stroke, more high-quality studies with random design should be conducted, particularly considering concealed allocation, blinded assessors, intent-to-treat analysis, and sample size calculation. Follow-up period should be considered after an intervention to determine the long-term effects of Baduanjin exercise for stroke rehabilitation. Isolating other treatments or co-intervention will allow for better explanation of the stand-alone benefits that Baduanjin exercise may have, as compared to either active or passive control. Finally, non-Chinese people with stroke should be investigated to determine if Baduanjin exercise is suitable and beneficial for other races with stroke.

## 5. Conclusions

Baduanjin exercise as a safe and adjunctive rehabilitation method might have beneficial effects for balance function, sensorimotor function of lower extremities, depression, the ability of daily activities, and quality of life in survivors of stroke. However, the small number of trials with methodological flaws in their design makes a definitive conclusion hard to be drawn. More well-designed randomized controlled trials with larger sample size and longer intervention periods are needed to establish the effects of Baduanjin on rehabilitative outcomes among stroke patients. In addition, strong scientific evidence could not be obtained because we could not eliminate bias: (1) It has not received international evaluation in the articles because these papers are all Chinese characters; (2) the authors are often overlapped eight extracted papers [20,23,24,26,27], and independent papers are regarded as three papers as research [21,22,25]; (3) the four selected studies [20,25,26,27] investigated the effects of Baduanjin exercise on balance function, as measured by BBS, two of the studies were conducted by a same research team. Based on the aforementioned limitations, at the present stage, there is publication bias. The bias is that published papers are limited to Chinese language only. There is also publication bias such as limitation of author, and few pieces of independent research. For reasons such as this, the effect of Baduanjin exercise is expected to be limited. At the present stage, because of the meta-analysis, although the effect was recognized, it is very weak as a scientific basis. Thus, the research on Baduanjin exercise will continue to accumulate.

## Figures and Tables

**Table 1:** Characteristics of studies selected in this systematic review.

Reference	Location (Language)	Participant Characteristics				Intervention Program	Outcome Measured	Results	Adverse Event	Follow-Up
		Sample Size (Dropout Rate)	Mean Age or Age Range	Course of Disease	Ischemic/Hemorrhage					
Bai et al. [20], RCT	Zhengzhou, China (Chinese)	60 (0%) BJ:30 CG:30	BJ:53.7 (4.5) CG:51.3 (7.5)	BJ:43.2 (6.53) day CG:38.5 (6.12) day	BJ:18/12 CG:19/11	BJ: 7 × 40 min/wk, 42 days + balance training CG: balance training	Balance (BBS)	BJ showed significantly greater improvement on balance performance than CG (*p* < 0.05)	No	No
Cai [21], RCT	Shanghai, China (Chinese)	60 (0%) BJ:30 CG:30	BJ:60.3 (10.5) CG:61.3 (7.4)	NR	BJ:21/9 CG:24/6	BJ: 4-to-5 × 30 min/wk, 3 months + educational lessons CG: educational lessons	Quality of life (WHOQOL)	Significant improvements on physical, psychological, and environment domains were only observed in BJ group (*p* < 0.05), but control group	No	No
Cai et al. [22], RCT	Shanghai, China (Chinese)	60 (0%) BJ:30 CG: 30	BJ:60.27 (10.48) CG:61.27 (7.42)	BJ:29.7 (7.38) wk CG:28.81 (5.37) wk	BJ:21/9 CG:24/6	BJ: 4-to-5 × 30 min/wk, 3 months + educational lessons CG: educational lessons	Activities of daily living (Barthel Scale)	Significant improvement in the Barthel Scale was only observed in BJ (*p* < 0.001), but control group	No	No
Chen et al. [23], RCT	Jinan, China (Chinese)	72 (0%) BJ:36 CG:36	BJ:52.21 (5.03) CG:51.83 (4.87)	BJ:5.83 (2.09) M CG:6.35 (1.69) M	BJ:20/16 CG:22/14	BJ1(CG):7 × 30 min/wk, 40 days + general rehabilitation BJ2: BJ + music therapy + general rehabilitation	Depression (HAMD) and quality of life (SF-36)	Both groups showed significant improvements in the two scales (*p* < 0.05) from baseline to post-test	No	No
Guo et al. [24], RCT	Zhengzhou, China, (Chinese)	224 (1.3%) BJ:115 CG:106	Age range from 33 to 82	Course of disease ranged from 1 to 6 months	NR	BJ: 7 × 40 min/wk, 6 weeks + (Bobath techniques and regular drug therapy); CG: Bobath techniques and regular drug therapy	Stroke-related neurologic deficit (NIHSS)	BJ showed significantly greater improvement in the NIHSS than control group (*p* = 0.001)	No	No
Tian [25], RCT	Qingdao, China (Chinese)	60 (5%) BJ:30 BJ:30	BJ:54.3 (4.7) CG:53 (4.3)	NR	60/0	BJ: 2 × 60–80 min/wk, 12 weeks (group-based self-practice) + general rehabilitation CG: general rehabilitation	Balance (BBS), sensorimotor function (FMA-LE)	BJ showed significantly greater improvement in both BBS and FMA-LE than control group (*p* < 0.01)	No	No
Zhang et al. [26], RCT	Zhengzhou, China, (Chinese)	224 (1.3%) BJ:115 CG:106	Age range from 33 to 82	Course of disease ranged from 1 to 6 months	NR	BJ: 7 × 40 min/wk, 6 weeks + (Bobath techniques and regular drug therapy); CG: Bobath techniques and regular drug therapy	Balance (BBS)	BJ showed significantly greater improvement in the BBS than control group (*p* < 0.01)	No	No
Zhang et al. [27], RCT	Jinan, China (Chinese)	62 (0%) BJ:31 CG:31	BJ:55.07 (4.81) CG:46.71 (3.57)	BJ: 6.22 (2.45) wk CG:7.01 (1.89) wk	BJ:21/10 CG:19/12	BJ:5 × 40 min/wk, 8 weeks + balance training + general rehabilitation CG: balance training + general rehabilitation	Sensorimotor function (FMA-LE) and balance (BBS)	BJ showed significantly greater improvements in the BBS and FMA-LE than control group (*p* < 0.05)	No	No

Note: wk = week; M = month; BJ = Baduanjin; CG = control group; BBS = The Berg Balance Scale; WHOQOL = World Health Organization Quality of Life; HAMD = Hamilton Depression Scale; SF-36 = 36-item Short Form Survey; NIHSS = National Institutes of Health Stroke Scale; FMA-LE = Fugl-Meyer Assessment Lower extremity; NR = Not Reported.

**Table 2:** Methodological quality for randomized controlled trials and non-randomized controlled studies.

Reference	Item 1	Item 2	Item 3	Item 4	Item 5	Item 6	Item 7	Item 8	Item 9	Item 10	Score
Bai et al. [20]	1	0	1	0	1	1	1	1	0	0	6/10
Cai [21]	1	0	1	1	1	1	1	1	0	0	7/10
Cai et al. [22]	1	0	1	1	1	1	1	1	0	0	7/10
Chen et al. [23]	1	0	1	1	1	1	1	1	0	0	7/10
Guo et al. [24]	1	0	1	0	1	0	1	1	0	0	5/10
Tian [25]	1	0	1	0	1	0	1	1	0	0	5/10
Zhang et al. [26]	1	0	1	0	1	0	1	1	0	0	5/10
Zhang et al. [27]	1	0	1	0	1	1	1	1	0	0	6/10

Note: Item 1 = randomization; Item 2 = concealed allocation; Item 3 = similar baseline; Item 4= blinding of assessors; Item 5 = more than 85% retention; Item 6 = missing data management (intention-to-treat analysis); Item 7 = between-group comparison; Item 8 = point measure and measures of variability; Item 9 = isolated Baduanjin intervention; Item 10 = prior sample size estimation;1 = explicitly described and present in details; 0 = absent, inadequately described, or unclear.
